# Lucio’s phenomenon, an uncommon occurrence among leprosy patients in Sri Lanka

**DOI:** 10.1186/s13104-015-1671-1

**Published:** 2015-11-13

**Authors:** Sandamalee Herath, Mitrakrishnan Rayno Navinan, Isurujith Liyanage, Nadeesha Rathnayaka, Jevon Yudhishdran, Janakie Fernando, Ganga Sirimanne, Aruna Kulatunga

**Affiliations:** National Hospital of Sri Lanka, Colombo, Sri Lanka; Department of Pathology, National Hospital of Sri Lanka, Colombo, Sri Lanka

**Keywords:** Hansens’ disease, Leprosy, Lucio’s, Lucio’s phenomenon

## Abstract

**Background:**

Lucio’s phenomenon is a rare manifestation of untreated leprosy which is seen almost exclusively in regions surrounding the Gulf of Mexico. Its occurrence elsewhere though documented is considered uncommon. We present a case of Lucio’s phenomenon in a previously undiagnosed leprosy patient who presented to us with its classical skin manifestations.

**Case presentation:**

A 64 year old South Asian (Sri Lankan) male with a history of chronic obstructive airway disease presented to us with fever and cough. He had a generalized smooth and shiny skin with ulcerating skin lesions afflicting the digits of the fingers. The lesions progressed to involve the extremities of the body and healed with crusting. Based on the clinical and investigational findings Tuberculosis and common vasculitic conditions were suspected and excluded. The unusual skin manifestations prompted a biopsy, and wade fite stained revealed *Mycobacterium* bacilli. In context of the clinical picture and histological findings, Lucio’s phenomenon was suspected. A clinical diagnosis of Lucio’s phenomenon occurring in the backdrop of lepromatous leprosy was made.

**Conclusion:**

Though leprosy is still a prevalent disease, it has manifestations that are not easily recognized or fully appreciated. Regional patterns of atypical manifestations should not limit better understanding of rarer manifestations as it will aid in clinching an early diagnosis and instituting prompt treatment, thereby reducing morbidity and mortality.

## Background

Hansen’s disease or leprosy is a chronic infectious disease caused by *Mycobacterium leprae*. The clinical course depends on the immune response mounted by the host. The presentation of infection shows a spectrum from tuberculoid to that of lepromatous versions of the illness [1]. Leprosy patients tend to have reactions based on the pattern of immune response elicited and depending on the mechanism these are further subcategorized. Lucio’s phenomenon is one such manifestation. First described in detail by Lucio and Alvarado and further elaborated by Latapi and Franken nearly a century later, Lucio’s phenomenon is a rare form of reaction seen in pure lepromatous leprosy (LL) or borderline lepromatous leprosy (BL) [2–4]. Some consider this entity a separate form of leprosy and define it as Lucio’s leprosy [5, 6] and attribute it to a newly discovered species, *M. lepromatosis* [7, 8]. Leprosy is still a globally prevalent disease [9], but seen in higher incidence in developing countries [10]. However Lucio’s phenomenon is a rare presentation seen exclusively in Mexico and Central America and is considered a globally restricted phenomenon [5, 11, 12]. But with increased recognition cases are being reported sporadically worldwide from South Asian and South East Asian countries [10, 13, 14], South Pacific-Polynesian islands [15], the Middle east and even African nations [9, 16–18] and thus should be suspected when clinically relevant even in non-endemic countries [19]. We present a rare occurrence of Lucio’s phenomenon, in a previously undiagnosed patient with lepromatous leprosy.

## Case presentation

**Fig. 1:**
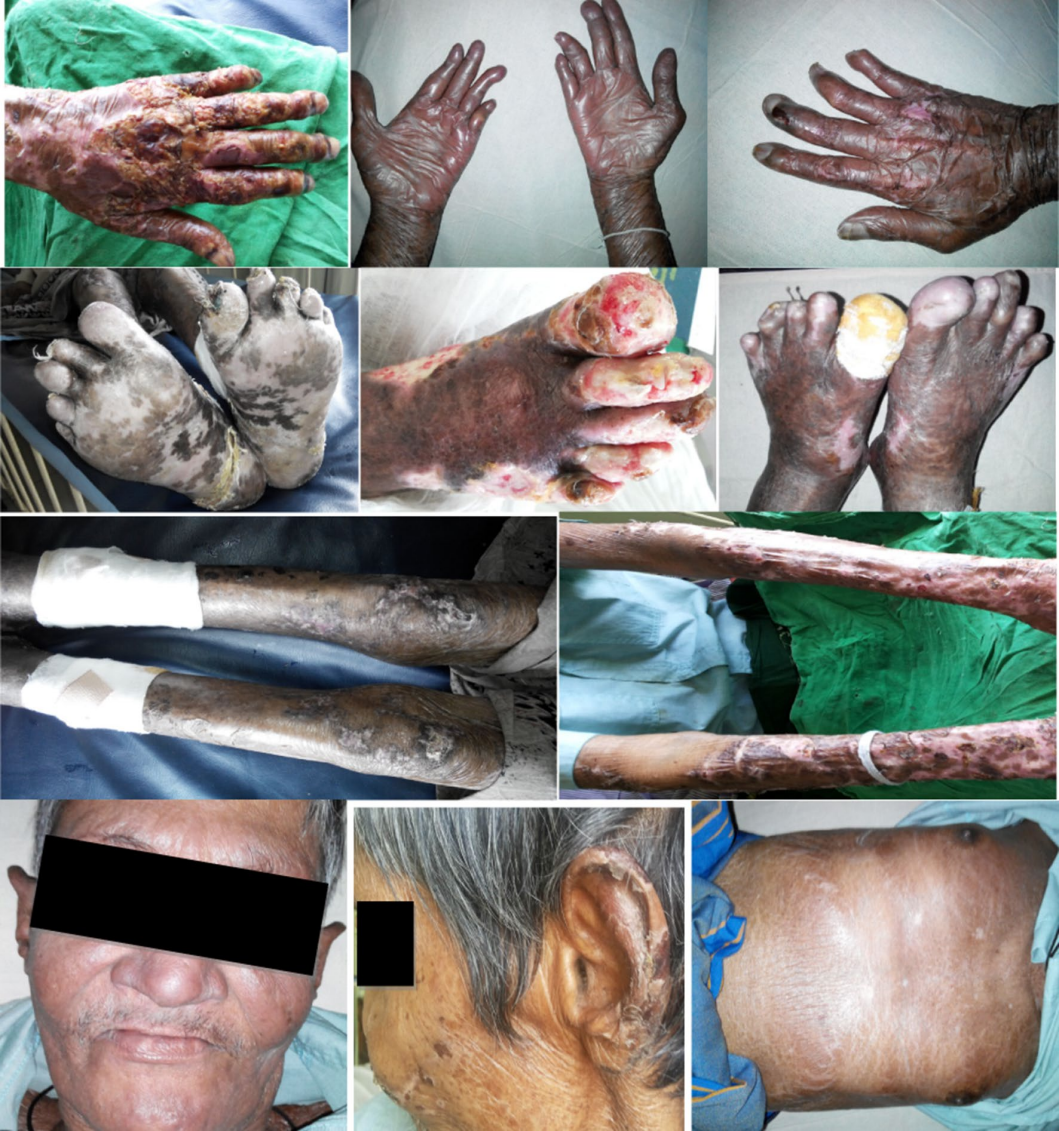
Skin manifestations of Lucio’s phenomenon observed. The first row depicts, bizarre shaped well demarcated superficial ulceration with slough formation (*left* image), which following treatment resulted in healing with cicatrization (*middle* and *right* images) The second row shows large denuded areas secondary to ulceration and necrosis of the foot (*left* and *middle*) finally resolving with cicatrization (*right* image). The third row depicts symmetrical involvement of the lower limbs (*left*) and upper limbs (*right*) with extensive bizarre ulceronecrosis. The fourth row depicts diffuse facial involvement with shiny skin, madarosis and infiltration with nodules in the chin (*left*) with involvement of the ear with necrotic lesions associated with crusting (*middle*) and mild truncal involvement with hairless shiny skin (*right*)

A 64 year old South Asian (Sri Lankan) man from a poor socio economic class who was previously diagnosed with chronic obstructive pulmonary disease (COPD) presented with an exacerbation of COPD with a productive cough of 2 weeks duration and recent onset fever. He also noted a recent swelling of both upper limbs that began during the same time period. He additionally complained of constitutional symptoms with loss of appetite with resultant loss of weight occurring over 6 month’s duration. He was a heavy smoker with 40 pack years, but has abstained for the last 5 years. He otherwise had no high risk behaviours and denied any prior history of joint, bowel or skin diseases. On general examination he was afebrile but clinically dehydrated and had pallor. His skin had a smooth sheen and felt very thin to touch. Both hands were oedematous and areas of necrosis were seen on the fingers. While warded in hospital his skin manifestations progressed. The lesions were seen predominantly below elbow and knees. He developed ulcerations, which were superficial demonstrating healthy granulation tissue, slough and areas of necrosis involving the dorsal surface of the extremities of both upper and lower limbs as well as the pinnae which healed with honey colored crusts (Fig. 1). Respiratory system examination revealed few bilateral crepitations. Examination of the abdominal, and cardiovascular systems failed to reveal significant abnormality. Neurological examination demonstrated a glove and stocking type peripheral sensory neuropathy for both soft touch and pain sensation. Similarly vibration was also noted to be diminished. Furthermore, bilateral thickening of the ulnar nerves were also noted on palpation.

**Fig. 2:**
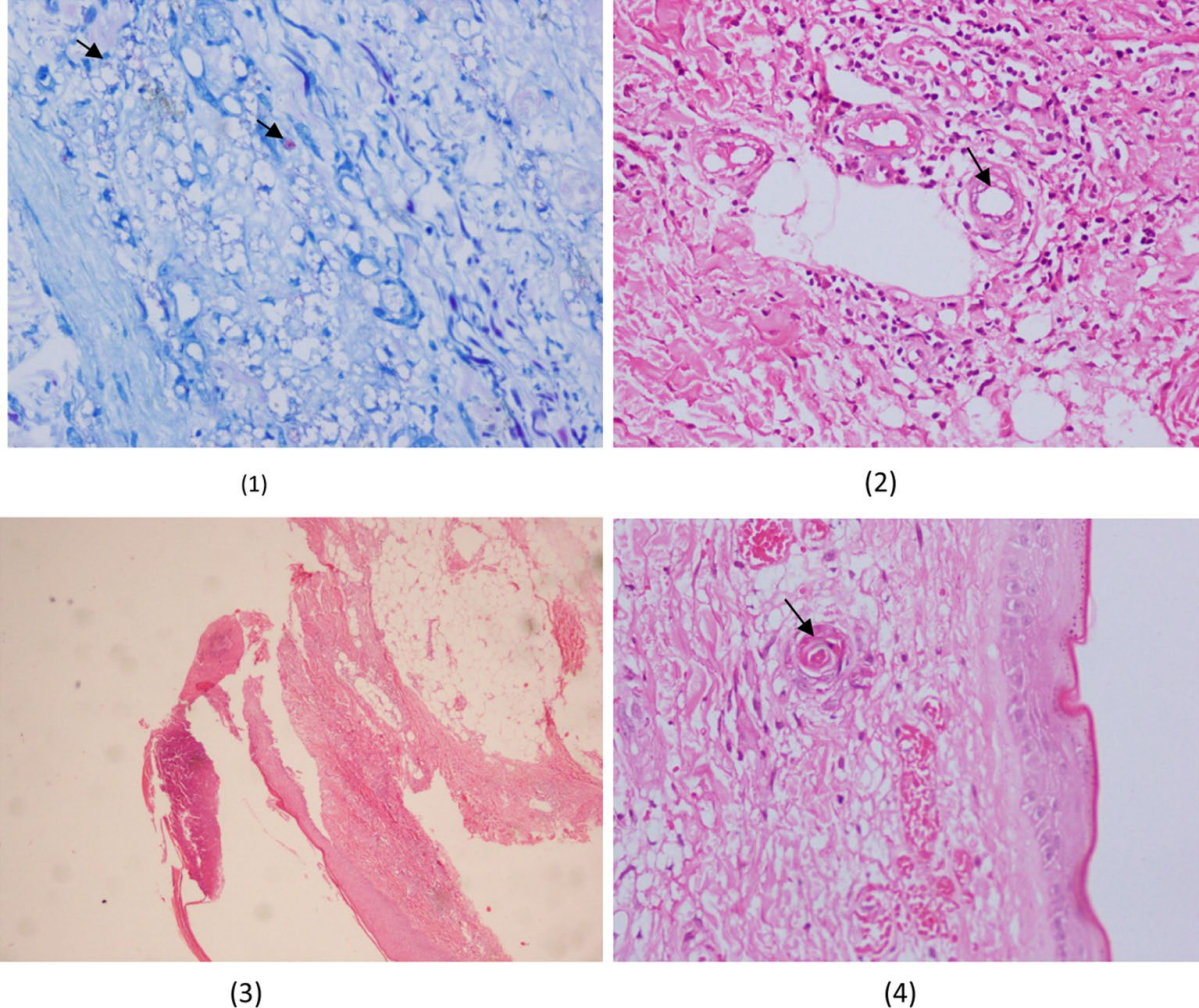
Histology of the skin biopsy. Image (*1*) depicts wade fite stain demonstrating the presence of (*red*) bacilli (indicated by *arrow*) within foamy cells and endothelial cells. Image (*2*) demonstrates blood vessels (indicated by *arrow*) with associated endothelial swelling. Image (*3*) shows presence of necrosis and ulceration of epidermis without panniculitis. Image (*4*) depicts endothelial proliferation and capillary thrombosis (indicated by *arrow*)

Whole blood analysis revealed severe anaemia with a haemoglogin of 5.9 g/dL (11–18) with a mean corpuscular volume (MCV) of 60 fL (80–100) and a mean corpuscular haemoglobin (MCH) of 17.8 pG (27–34). White cell count (WCC) was normal with a value of 9.8. × 10^9^/L (4–10) with a predominant neutrophillic count of 80 %. Platelets were elevated to a value of 522 × 10^9^/L (150–450). Blood picture reflected the whole blood analysis with toxic changes seen with a neutrophil leukocytosis favouring an ongoing bacterial infection and reactive thrombocytosis. Iron studies revealed reduced serum iron with a value of 15 µg/dL (41–132) with reduced iron saturation of 7.8 % (15–35) and a reduced total iron binding capacity of 192 µg/dL (228–428) suggestive of anaemia of chronic disease. Serum ferritin was elevated at 539 ng/ml (20–400). Preliminary liver enzymes were normal as was the bilirubin level. Serum proteins were elevated with a value of 88 g/L (61–77) with a predominant globulin fraction with 53 g/L (22–40). Serum protein electrophoresis demonstrated marked poly-clonal gammopathy with prominent alpha 1 & 2 bands and markedly diminished albumin levels. Erythrocyte sedimentation rate (ESR) was very high with a value of 149 mm for the 1st hour (<15) and similarly C-reactive protein was elevated to a value of 167 mg/L (<8). Serum lactate dehydrogenase was normal with a value of 260 U/L (230–460). Fasting blood sugar was normal. Sputum was checked for acid fast bacilli (AFB) as the patient presented with a chronic cough and it revealed the presence of abundant acid fast bacilli although sputum for *Mycobacterium tuberculosis* polymerase chain reaction (PCR) and culture were negative. Imaging of the chest with plain chest X-ray and high resolution contrast CT were failed to detect significant abnormality despite the positive acid fast bacilli found in sputum. Anti-nuclear antibodies, anti-neutrophil cytoplasmic auto antibodies P and C, rheumatoid factor, cryoglobulin were all negative and complement 3 and complement 4 levels were normal. VDRL, Hepatitis B surface antigen, Hepatitis C antibodies and human immunodeficiency virus antibody I and II were negative. Blood and urinary cultures failed to reveal a growth. Upper gastrointestinal endoscopy revealed erosive esophagitis with multiple small ulcers all over the body of the stomach and the presence of duodenitis. Skin biopsy revealed clusters of foamy macrophages within the dermis and wade fite stained positive for *Mycobacterium* bacilli. Focal necrosis was also seen in the epidermis. A slit skin smear showed a bacillary index of +4 (indicating at least 10 bacilli in every field).The histology demonstrated the presence of thrombosed capillary vessels in the absence of inflammatory cells, though panniculitis was not noted on the analyzed specimen, in context with the clinical picture Lucio’s leprosy was favored as the diagnosis (Fig. 2). PCR for *M. tuberculosis* of the bone marrow was negative. A clinical diagnosis of Lucio’s phenomenon secondary to lepromatous leprosy was made and multi drug therapy regimen was started. As the patient had already been transfused with blood since he was severely anaemic and emaciated Glucose-6-phosphate-dehydragenase (G6PD) enzyme levels were not checked and dapsone was withheld from the treatment regimen. Thus the patient was started on daily doses of clofazimine with ofloxacin together with monthly high dose rifampicin and clofazimine according to guidelines on the management of lepromatous leprosy. The patient developed secondary skin infection in the affected areas, with resultant fever. In ward therapy was carried out till patient showed clinical improvement. Frequent cleaning and dressing of the ulcerated areas of the limbs and digits were done under antibiotic cover. Supportive therapy was also incorporated with high protein and caloric diet along with intravenous albumin infusion along with rehabilitation and physiotherapy. The patient demonstrated satisfactory response to treatment with resolution of secondary local sepsis and visible clinical recovery was also seen with the onset of healing of ulcers due to Lucio’s phenomenon.

## Discussion

The patient in this case study presented with fever and in the background of COPD the initial working diagnosis was that of a respiratory tract infection complicating COPD. However a high ESR with a history of chronic cough in a country endemic to tuberculosis, prompted us to exclude pulmonary tuberculosis (PTB). Though our suspicion was strengthened by the sputum analysis demonstrating AFB, the negative PCR analysis and culture along with the lack findings favoring PTB on chest imaging caused clinical confusion. Furthermore our initial assumption of a dual pathology for his skin manifestation due to vasculitic type lesions prompted further investigations to exclude usual causative agents with serological studies, and biopsy was taken akin for this purpose. Though leprosy was entertained in the differential diagnosis the obscure nature of this specific manifestation did not immediately direct us towards its diagnosis. But the skin biopsy helped us finally clinch a unifying diagnosis in context of the clinical presentation of Lucio’s phenomenon secondary to lepromatous leprosy. Though he never had continuous follow up for COPD, the lack of awareness among the general populace and possibly clinicians who may have been consulted previously is demonstrated by the late identification of his advanced state of leprosy. This goes to demonstrate that despite being a country afflicted by leprosy its rarer manifestations are still not fully appreciated.

Lucio’s phenomenon is a reactional state and is thought to occur in the lepromatous version of the illness where the unresponsiveness of the immune system allows unrestricted proliferation of *Mycobacterium lepromatosis* sp. nov, a newly discovered species with traits that allow it to manifest distinct clinic-pathological features more towards the diffuse end of the spectrum of the disease [2, 7, 8, 19, 20]. Lucio’s phenomenon may present as a new feature in an already well-established, yet untreated leprosy patient. It can also be the preliminary symptom introducing the lepromatous version of the disease. Rarely, it can present in an already fully treated diffuse lepromatous leprosy patient in the absence of bacilli, even years later [4, 5, 15, 16].

The cutaneous manifestations of Lucio’s may be difficult to identify as the skin manifestations tend to be diffuse and variable. But its propensity to cause significant morbidity and scarring with possible fatal outcome stresses the importance of early identification of this treatable condition [5, 21]. The skin manifestations classically occur in the absence of constitutional symptoms and include generalized dryness and shininess, with a myxedematous appearance, thus being called “leprabonita or pretty leprosy”. There may also be hair loss with resultant alopecia involving the eyebrows, eyelashes and other areas of body hair. The ongoing vasculitic process causes either defined and or irregular erythematous spots with painless hemorrhagic blisters. These can evolve into necrotic areas with scabs and ulcerate with angular margins causing pain. Though there is a predilection for the extremities, the affected area can be diffuse and involve the trunk and the face [12, 19]. The lesions can recur and have a destructive effect especially when afflicting superficial regions of the body e.g., pinna, nose. The disease process may involve the subcutaneous tissue due to the erosive process and cause significant scarring. If the involvement is severe the ischaemic necrosis of the skin can result in detachment [2, 4, 12, 16, 21–25]. Though fever may not be part of primary spectrum of the disease, secondary sepsis may herald its onset. Our patient in hindsight demonstrated most of these classical manifestations and pattern of presentation (Fig. 1).

A commonly encountered problem is that Lucio’s may be confused with erythema nodosum with necrosis, which Bernard and colleagues help differentiate [24]. Accordingly, our patients skin manifestations occurred in the pure primitive and diffuse form of leprosy, prior to initiating treatment with typical irregularly shaped superficial ulcers followed by crusting, clinically favored Lucio’s more than erythema nodosum with necrosis. Furthermore, histologically the absence of inflammatory infiltrate together with the presence of thrombosed vessels of the superficial dermis associated with necrosis also favored Lucios. The presence of fever was atypical, but maybe attributable to a respiratory infection complicating COPD. Thus, based on the classical skin manifestation, the advanced stage of the disease and the histological findings of thrombosed vessels in the absence of neutrophillic infiltrates (Fig. 2) our patients’ diagnosis favors that of Lucio’s.

Early diagnosis and prompt treatment is beneficial as it can improve outcome [14]. Overall response to treatment may be seen rapidly, between 1 week to 1 month following initiation of microbicidal drug treatment [26]. The pure form of Lucio’s may only necessitate the usual multi drug regimen. Thus making the usual anti-leprosy drugs an effective first line treatment [15] without requiring thalidomide or systemic corticosteroids to be included into the regimen [6, 27], though these have also been opted to be used in severe situations and found useful [21]. However, fatality has been seen despite aggressive treatment [28]. This is attributable to secondary infection and sepsis due to the presence of a susceptible of skin condition, poor nutritional status, opted treatment regimens [19] and delay in diagnosis or misdiagnosis [22].

Our patient required an urgent blood transfusion prior to a definitive diagnosis, thus G6PD levels were not checked as the lab interpretation would not be accurate, so to err on the side of caution the patient was not initiated on dapsone. Instead ofloxacin was substituted along with the remainder of the multi-bacillary regimen in accordance to guidelines on treatment. Supportive measures were undertaken with the institution of high protein diet, rehabilitation and physiotherapy. Frequent wound dressing along with the initiation of antibiotics to control secondary infection helped quicken the resolution. The patient showed improvement with treatment and this was sustained on review a month following discharge from hospital (Fig. 1).

## Conclusion

Though leprosy is not uncommon, Lucio’s is a rare presentation. If encountered in the absence of familiarity, diagnosis may be unduly delayed. Clinicians should be wary of even rare presentations of commonly encountered diseases like leprosy despite their uncommon incidence due to regional patterns of prevalence. Lack of awareness may be the reason why phenomena such as Lucio’s are under diagnosed, unreported and remain in obscurity.

## Consent

Written informed consent was obtained from the patient for publication of this Case Report and any accompanying images.

## Abbreviations

LL: lepromatous leprosy
BL: borderline leprosy
AFB: acid fast bacilli
G6PD: glucose-6-phosphate-dehydragenase
PCR: polymerase chain reaction
PTB: pulmonary tuberculosis
ESR: erythrocyte sedimentation rate
COPD: chronic obstructive pulmonary disease
